# Assessment of Diagnosis and Triage in Validated Case Vignettes Among Nonphysicians Before and After Internet Search

**DOI:** 10.1001/jamanetworkopen.2021.3287

**Published:** 2021-03-29

**Authors:** David M. Levine, Ateev Mehrotra

**Affiliations:** 1 Division of General Internal Medicine and Primary Care, Brigham and Women’s Hospital, Boston, Massachusetts; 2 Harvard Medical School, Boston, Massachusetts; 3 Department of Health Care Policy, Harvard Medical School, Boston, Massachusetts; 4 Division of General Medicine and Primary Care, Beth Israel Deaconess Medical Center, Boston, Massachusetts

## Abstract

**Importance:**

When confronted with new medical symptoms, many people turn to the internet to understand why they are ill as well as whether and where they should get care. Such searches may be harmful because they may facilitate misdiagnosis and inappropriate triage.

**Objective:**

To empirically measure the association of an internet search for health information with diagnosis, triage, and anxiety by laypeople.

**Design, Setting, and Participants:**

This survey study used a nationally representative sample of US adults who were recruited through an online platform between April 1, 2019, and April 15, 2019. A total of 48 validated case vignettes of both common (eg, viral illness) and severe (eg, heart attack) conditions were used. Participants were asked to relay their diagnosis, triage, and anxiety regarding 1 of these cases before and after searching the internet for health information.

**Exposures:**

Short, validated case vignettes written at or below the sixth-grade reading level randomly assigned to participants.

**Main Outcomes and Measures:**

Correct diagnosis, correct triage, and flipping (changing) or anchoring (not changing) diagnosis and triage decisions were the main outcomes. Multivariable modeling was performed to identify patient factors associated with correct triage and diagnosis.

**Results:**

Of the 5000 participants, 2549 were female (51.0%), 3819 were White (76.4%), and the mean (SD) age was 45.0 (16.9) years. Mean internet search time was 12.1 (95% CI, 10.7-13.5) minutes per case. No difference in triage accuracy was found before and after search (74.5% vs 74.1%; difference, −0.4 [95% CI, −1.4 to 0.6]; *P* = .06), but improved diagnostic accuracy was found (49.8% vs 54.0%; difference, 4.2% [95% CI, 3.1%-5.3%]; *P* < .001). Most participants (4254 [85.1%]) were anchored on their diagnosis. Of the 14.9% of participants (n = 746) who flipped their diagnosis, 9.6% (n = 478) flipped from incorrect to correct and 5.4% (n = 268) flipped from correct to incorrect. The following groups had an increased rate of correct diagnosis: adults 40 years or older (eg, 40-49 years: 5.1 [95% CI, 0.8-9.4] percentage points better than those aged <30 years; *P* = .02), women (9.4 [95% CI, 6.8-12.0] percentage points better than men; *P* < .001), and those with perceived poor health status (16.3 [95% CI, 6.9-25.6] percentage points better than those with excellent status; *P* = .001) and with more than 2 chronic diseases (6.8 [95% CI, 1.5-12.1] percentage points better than those with 0 conditions; *P* = .01).

**Conclusions and Relevance:**

This study found that an internet search for health information was associated with small increases in diagnostic accuracy but not with triage accuracy.

## Key Points

**Question:**

Is there an association between an internet search for health information and improved accuracy in diagnosis and triage among nonphysicians?

**Findings:**

In this survey study of 5000 US adults who were asked to assess validated case vignettes, small improvements in diagnostic accuracy were found after an internet search for health information, but no difference in triage accuracy was observed. Adults 40 years or older, women, and those with poor health status were superior at diagnosis.

**Meaning:**

Results of this study suggest that, contrary to concerns of its harmfulness, an internet search was associated with modest improvements in diagnosis but had no association with triage.

## Teaser

This survey study evaluates the factors associated with accurate diagnosis and triage by nonphysician adults who use the internet to find health information.

## Introduction

Each day, millions of people worldwide who are confronted with new medical symptoms turn to the internet before seeking care to understand why they are ill, whether they should get care, and where they should get care.^1,2^ The value of performing an internet search for health purposes is controversial, with concerns that it leads to inaccurate diagnosis, inappropriate triage (ie, choosing the right location to seek care), and increased anxiety (cyberchondria).^3,4,5,6,7^ An internet search may lead people to low-quality health information that might hurt their choice of whether to get care or to alarming content that might easily overwhelm or confuse people. Some governments have even launched Don’t Google It advertising campaigns to urge their residents to not use the internet to search their health concerns.^8,9^

Despite its ubiquitous use, the benefits and harms of an internet search for health information are poorly understood. Previous research has been largely limited to observational studies of internet search behavior and may lack a criterion standard.^2,10,11,12^ In this study, we sought to empirically measure the association of an internet search with diagnosis, triage, and anxiety by presenting laypeople with a clinical vignette and assessing the accuracy of their decisions before and after searching the internet.

## Methods

We performed a before-after survey study with a national sample of internet users in the United States. Participants reviewed a simple case vignette and relayed their presumed diagnosis, triage, and anxiety regarding the case. Next, participants were asked to use the internet to search for information about the case and relay their updated diagnosis, triage, and anxiety. This study design emulated how a person typically interacts with the internet: encountering information, forming a preliminary conclusion, and then reforming a conclusion after searching the internet. We enrolled participants between April 1, 2019, and April 15, 2019, and conducted no follow-up. Participants provided written informed consent by clicking on the accept button in the online survey after reading a description of the study and its risks and benefits. The protocol was approved by the Harvard Medical School Institutional Review Board. We followed the American Association for Public Opinion Research (AAPOR) reporting guideline.

### Setting and Participants

We recruited people using Toluna, a company specializing in online surveys for research, marketing, and business intelligence. Toluna uses a multifaceted approach to recruit a nationally representative sample of survey respondents. We requested a representative cohort by sex, age, and census region. Toluna also deploys multiple quality assurance strategies as respondents complete a survey to ensure high-quality data. People were eligible for inclusion in this survey if they were 18 years or older and resided in the United States. Participants earned compensation for their time through Toluna’s standard reimbursement system.

Separately from the online survey participants, we enrolled a convenience sample of 21 attending primary care physicians at Harvard Medical School to validate the case vignettes. Each physician received a $20 gift card for their time.

The correct diagnosis and triage category for each vignette were first ascertained by the 2 of us (both general internists) and used as the criterion standard. We gave the primary care physicians the 48 clinical vignettes and asked for their triage and diagnosis on the basis of only their clinical experience. The physicians did not use the internet.

### Intervention and Outcomes

Building on prior work that evaluated online symptom checker tools,^13,14^ we created 48 case vignettes that included a chief complaint followed by additional pertinent details (eTable 1 in the Supplement). Each vignette was fewer than 50 words and written at or below a sixth-grade reading level. Twelve vignettes were written for each of 4 triage categories: emergent cases, same-day cases, 1-week cases, and self-care cases. We included both common (eg, viral illness) and severe (eg, heart attack) conditions but not those with highly obscure presentations.

Before beginning the principal task, respondents were asked to report on several sociodemographic variables (Table 1). Respondents were randomly assigned to 1 of the 48 vignettes and given instructions to “please read the following health problem, and imagine it were happening to your close family member.” After reviewing the vignette, participants selected from the following triage options that they deemed the best: (1) let the health issue get better on its own; the issue most likely does not require seeing a doctor; (2) try to see a doctor within a week; the issue likely will not get better on its own, but it is also not an emergency; (3) try to see a doctor within a day; the issue is urgent, but it is not an emergency; or (4) call 911 or go directly to the emergency department; the issue requires immediate attention.

**Table 1.** Participant Characteristics

Characteristic	Participants, No. (%)^a^
Total participants, No.	5000
Age, mean (SD), y	45.0 (16.9)
Female sex	2549 (51.0)
Male sex	2451 (49.0)
Race/ethnicity	
Non-Hispanic White	3819 (76.4)
Non-Hispanic Black	404 (8.1)
Hispanic	318 (6.4)
Non-Hispanic Asian	309 (6.2)
Non-Hispanic other or multiple	150 (3.0)
Census region	
Northeast	950 (19.0)
Midwest	1150 (23.0)
South	1750 (35.0)
West	1150 (23.0)
Married or partnered	2848 (57.0)
Educational level	
<High school diploma	103 (2.1)
High school diploma or GED certificate	1047 (20.9)
Some college	1709 (34.2)
Bachelor's degree	1435 (28.7)
>Bachelor's degree	706 (14.1)
Health insurance coverage	
Uninsured	443 (8.9)
Medicare	1147 (22.9)
Medicaid	535 (10.7)
Both Medicare and Medicaid	187 (3.7)
Private or employer-based	2484 (49.7)
Not sure	204 (4.1)
Perceived health status	
Excellent	690 (13.8)
Very good	1780 (35.6)
Good	1794 (35.9)
Fair	605 (12.1)
Poor	131 (2.6)
Employed	2940 (58.8)
Family income, US $	
<30 000	1260 (25.2)
30 000-49 999	991 (19.8)
50 000-79 999	1182 (23.6)
80 000-99 999	534 (10.7)
100 000-149 999	660 (13.2)
150 000-199 999	225 (4.5)
≥200 000	148 (3.0)
Chronic disease, No.	
0	2612 (52.2)
1	1066 (21.3)
2	617 (12.3)
>2	520 (10.4)
Not sure	185 (3.7)
Has primary care	3963 (79.3)
Visits in past 6 mos, mean (95% CI)	
Physician visit	2.1 (2.0-2.1)
ED visit	0.3 (0.3-0.3)
Hospital admissions in past 6 mos, mean (95% CI)	0.2 (0.2-0.2)
Global health care rating in past 6 mos, mean (95% CI), 0-10 points	7.4 (7.3-7.5)

Abbreviations: ED, emergency department; GED, general educational development.

^a^ Nationally representative sample by age, sex, and census region. Percentages may not sum to 100 because of rounding.

Next, via free-text response to the following question, participants listed the conditions in order of likelihood: “What do you think are the three most likely medical diseases or diagnoses that could be causing this health problem?” Using a Likert scale (not at all, slightly, moderately, highly, or extremely), they selected an emotional response to this question: “If you had a family member experience the health problem described, how nervous/anxious/on edge would you be?”^15^ In addition, they were asked to rate their confidence in their responses using the same Likert scale.

Participants were then asked to use the internet in any way they believed to be useful to find the correct diagnosis and triage option for the health problem in the same vignette. We measured the time spent searching in minutes. After the internet search, respondents reported the triage and diagnosis that they selected and, using the same Likert scale, ranked their level of anxiety and confidence in their response as well as their perceived difficulty in finding useful information, their trust in the information they found, and the kinds of websites that they deemed to be most helpful. We categorized websites as a search engine, health specialty site (for example, WebMD), general information site (for example, Wikipedia), social network site (for example, Facebook), news site, forum, or other.

### Statistical Analysis

We presented descriptive data with counts and percentages or means and 95% CIs, as appropriate. Because of the survey design, there were no missing data. Free-text diagnoses were manually reviewed and scored. Consistent with prior work,^13,14^ we measured diagnostic accuracy in 2 ways: whether the respondent’s first selected diagnosis was correct (first correct) and whether any of the respondent’s 3 diagnoses were correct (any correct). In this article, we present the analyses of any-correct diagnosis, whereas eTable 2 in the Supplement presents first-correct analyses.

We used 2 methods to assess the accuracy of the triage: whether the respondent’s selected triage was exactly correct (exact) and whether the respondent’s selected triage matched a dichotomized triage variable of emergent or same-day cases vs 1-week or self-care cases (dichotomized). In the main analyses, we present a dichotomized triage as we observed some inconsistency among physicians in triage with emergent vs same-day cases^16^; the exact triage analyses are included in eTable 2 in the Supplement.

To identify whether acuity was associated with diagnosis or triage, we performed subgroup analysis by acuity. We assessed flipping (changing the answer) and anchoring (not changing the answer) for both diagnosis and triage decision.

For a bivariate comparison of diagnosis and triage, we used the McNemar test, and for anxiety and confidence, we used a paired, 2-tailed *t* test. Diagnosis and triage stratified by participant characteristics are shown in eTables 3 and 4 in the Supplement. For multivariable analyses, we used general estimating equations that accounted for repeated measures. We present marginal effects for associations for values of the independent variable of interest while adjusting for all covariates in Table 1. Adjusted odds ratios are presented in eTable 5 in the Supplement.

We considered 2-sided *P* < .05 to be significant. We performed all analyses in SAS, version 9.4 (SAS Institute Inc), and Stata, version 15 (StataCorp LLC).

## Results

We enrolled 5000 participants with a mean (SD) age of 45.0 (16.9) years. Of these participants, 2549 were female (51.0%), 3819 were White (76.4%), 2484 were privately insured (49.7%), 736 had poor or fair perceived health (14.7%), and 2203 had at least 1 chronic disease (44.1%) (Table 1). Most participants (3963 [79.3%]) reported having primary care. Respondents reported a mean of 2.1 (95% CI, 2.0-2.1) physician visits and 0.3 (95% CI, 0.3 to 0.3) emergency department visits in the past 6 months.

The 21 primary care physicians who validated the correct responses from the clinical vignettes included 10 women (47.6%) and 11 men (52.4%) with a mean duration of practice of 12.4 years. The physicians reported the correct triage in 91.2% (95% CI, 89.2%-93.2%) of vignettes and the correct diagnosis in 95.7% (95% CI, 94.3%-97.0%) of vignettes.

### Before Internet Search

**Figure 1.**
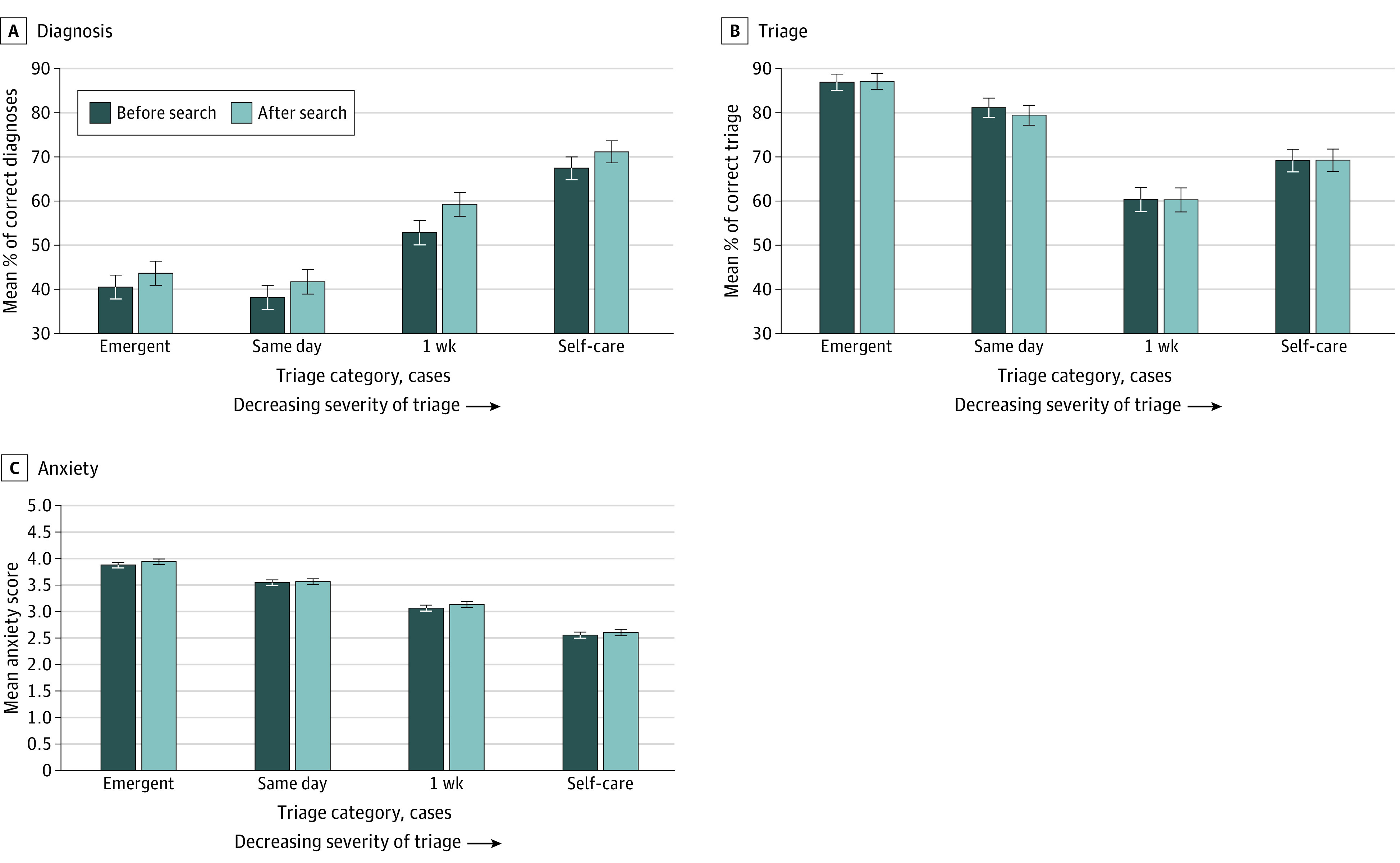
Diagnosis, Triage, and Anxiety Before and After an Internet Search, by Severity of Case Error bars represent 95% CI. For panels A and B, the y-axis does not begin at 0.

Before conducting an internet search, respondents differed in diagnosis, triage, and anxiety across triage categories (Figure 1; eTable 6 in the Supplement). Rates of correct diagnosis for emergent cases (40.4%; 95% CI, 37.8%-43.2%) were significantly lower than for self-care cases (67.4%; 95% CI, 64.8%-70.0%) (Figure 1A). The opposite was true for triage, where rates of correct triage for emergent cases (87.0%; 95% CI, 85.2%-88.9%) were significantly higher than for self-care cases (69.3%; 95% CI, 66.7%-71.8%) (Figure 1B). As the case acuity became more serious, respondents reported more anxiety (on a 5-point scale: self-care cases, 2.6 of 5; emergent cases, 3.9 of 5) (Figure 1C).

### After Internet Search

**Figure 2.**
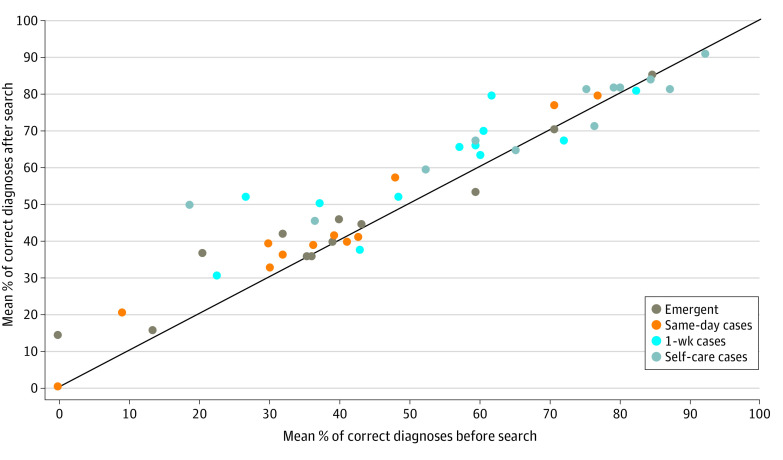
Diagnostic Accuracy Before and After Internet Search, by Severity of Case

Respondents spent a mean internet search time of 12.1 (95% CI, 10.7-13.5) minutes per case. There was a significant increase in diagnostic accuracy observed before vs after the internet search (49.8% vs 54.0%; difference, 4.2% [95% CI, 3.1%-5.3%]; *P* < .001) (Figure 1A). Improvements in diagnostic accuracy were observed across all triage categories: emergent (3.1%; 95% CI, 1.0%-5.3%; *P* = .004), same-day (3.5%; 5% CI, 1.5%-5.6%; *P* < .001), 1-week (6.4%; 95% CI, 4.1%-8.7%; *P* < .001), and self-care (3.7%; 95% CI, 1.7%-5.8%; *P* < .001) cases (Figure 2; eTable 6 in the Supplement).

No difference in triage accuracy was observed between before and after the internet search across all cases (74.5% vs 74.1%; difference, −0.4 [95% CI, −1.4 to 0.6]; *P* = .06) or among any level of case acuity (Figure 1B; eTable 6 in the Supplement). Anxiety also did not change from before to after search (3.3 of 5 points vs 3.3 of 5 points) (Figure 1C; eTable 6 in the Supplement), nor did participants’ confidence in their responses (3.8 of 5 points vs 3.8 of 5 points) (eTable 6 in the Supplement).

Participants reported that, in general, it was slightly difficult to find useful information on the internet and they moderately trusted the information found (eTable 7 in the Supplement). They noted that the most helpful sources of information were search engines (48.2% [n = 2411]), followed by health specialty sites (42.9% [n = 2145]). A small proportion of respondents (1.5% [n = 73]) rated social network sites as most helpful.

### Anchoring and Flipping

**Figure 3.**
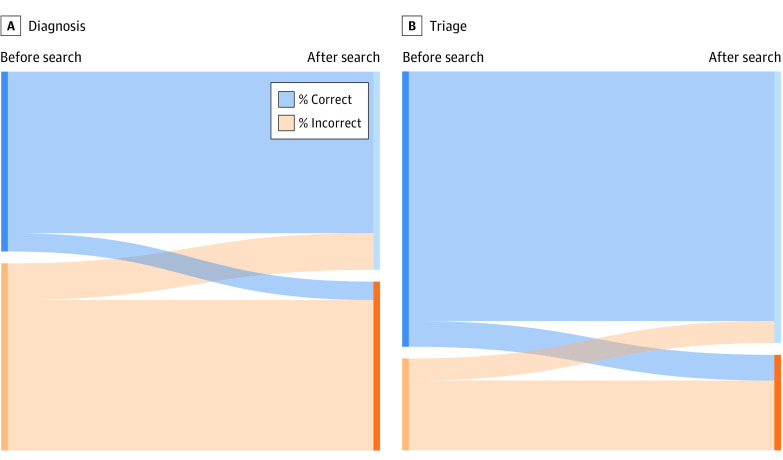
Anchoring or Flipping on Original Diagnosis and Triage The intersection of blue and orange color blocks represents the percentage of patients who flipped their original diagnosis (A) or triage (B).

Most respondents were anchored on their original diagnosis (4254 [85.1%]) or triage (4360 [87.2%]) (Figure 3; eFigure in the Supplement). A small proportion (14.9% [n = 746]) flipped their diagnosis after the internet search: 9.6% (n = 478) changed from incorrect to correct diagnosis, whereas 5.4% (n = 268) changed from correct to incorrect diagnosis. Similarly, 12.8% of respondents (n = 640) flipped their triage decision after the internet search, with roughly similar percentages in both directions: 6.6% (n = 329) changed from correct to incorrect triage, whereas 6.2% (n = 311) changed from incorrect to correct triage.

### Factors Associated With Correct Diagnosis and Triage

In multivariable modeling, characteristics that were associated with an increased rate of correct diagnosis were age of 40 years or older (40-49 years: 5.1 [95% CI, 0.8-9.4] percentage points more than for those <30 years [*P* = .02]; 50-59 years: 6.6 [95% CI, 1.8-11.3] percentage points more than for those <30 years [*P* = .006]), female sex (9.4 [95% CI, 6.8-12.0] percentage points more than for male sex; *P* < .001), White race/ethnicity (9.6 [95% CI, 4.9-14.4] percentage points more than for Black race/ethnicity [*P* < .001]; 6.4 [95% CI, 0.9-11.8] percentage points more than for Hispanic race/ethnicity [*P* = .02]; and 14.7 [95% CI, 9.3-20.0] percentage points more than for Asian race/ethnicity [*P* < .001]), uninsured status (9.1 [95% CI, 3.2-15.0] percentage points more than for Medicare; *P* = .003), perceived poor health status (16.3 [95% CI, 6.9-25.6] percentage points higher than for those with excellent status; *P* = .001), and more than 2 chronic diseases (6.8 [95% CI, 1.5-12.1] percentage points higher than for those with 0 conditions; *P* = .01) (Table 2).

**Table 2.** Associations of Sociodemographic Variables With Any Correct Diagnosis and Dichotomized Triage Before and After Internet Search

Variable	Correct diagnosis		Correct triage	
	Marginal effect size (95% CI)^a^	*P* value	Marginal effect size (95% CI)^a^	*P* value
Age, y^b^				
<30	1 [Reference]		1 [Reference]	
30-39	1.0 (−3.0 to 5.1)	.63	0.5 (−3.1 to 4.0)	.80
40-49	5.1 (0.8 to 9.4)	.02	2.9 (−0.8 to 6.7)	.13
50-59	6.6 (1.8 to 11.3)	.006	5.9 (1.8 to 10.0)	.005
60-69	4.1 (−1.5 to 9.7)	.15	2.3 (−2.7 to 7.3)	.36
≥70	5.3 (−1.7 to 12.3)	.14	3.5 (−2.4 to 9.5)	.25
Sex				
Female	1 [Reference]		1 [Reference]	
Male	−9.4 (−12.0 to −6.8)	<.001	−4.5 (−6.8 to −2.3)	<.001
Race/ethnicity				
Non-Hispanic White	1 [Reference]		1 [Reference]	
Non-Hispanic Black	−9.6 (−14.4 to −4.9)	<.001	−9.7 (−14.2 to −5.2)	<.001
Hispanic	−6.4 (−11.8 to −0.9)	.02	−2.9 (−7.4 to 1.6)	.21
Non-Hispanic Asian	−14.7 (−20.0 to −9.3)	<.001	−2.0 (−6.7 to 2.7)	.41
Non-Hispanic other or multiple	0.4 (−7.2 to 7.9)	.93	−0.2 (−6.6 to 6.2)	.94
Census region				
Northeast	1 [Reference]		1 [Reference]	
Midwest	0.5 (−3.4 to 4.3)	.82	−0.5 (−4.0 to 3.0)	.78
South	0.7 (−2.9 to 4.2)	.72	1.2 (−2.0 to 4.3)	.47
West	−1.1 (−5.0 to 2.8)	.58	−3.0 (−6.5 to 0.5)	.09
Partner status				
Married or partnered	1 [Reference]		1 [Reference]	
Not married or partnered	0.4 (−2.4 to 3.2)	.78	0.5 (−2.0 to 2.9)	.72
Educational level				
<High school diploma	1 [Reference]		1 [Reference]	
High school diploma or GED certificate	1.0 (−8.1 to 10.1)	.83	3.2 (−5.0 to 11.4)	.45
Some college	4.9 (−4.1 to 14.0)	.28	6.2 (−1.9 to 14.3)	.14
Bachelor's degree	5.5 (−3.8 to 14.7)	.25	6.2 (−2.1 to 14.5)	.14
>Bachelor's degree	7.3 (−2.4 to 16.9)	.14	6.9 (−1.8 to 15.6)	.12
Health insurance coverage				
Uninsured	1 [Reference]		1 [Reference]	
Medicare	−9.1 (−15.0 to −3.2)	.003	−3.6 (−8.7 to 1.5)	.17
Medicaid	−3.0 (−8.9 to 2.9)	.32	−4.4 (−9.6 to 0.7)	.09
Both Medicare and Medicaid	−9.9 (−18.2 to −1.5)	.02	−7.5 (−15.0 to −0.1)	.048
Private or employer-based	−0.1 (−5.3 to 5.0)	.96	1.0 (−3.4 to 5.3)	.66
Not sure	−6.2 (−14.0 to 1.6)	.12	−7.4 (−14.3 to −0.4)	.04
Perceived health status				
Excellent	1 [Reference]		1 [Reference]	
Very good	7.1 (3.1 to 11.2)	.001	1.0 (−2.6 to 4.6)	.58
Good	8.9 (4.6 to 13.2)	<.001	5.0 (1.2 to 8.8)	.01
Fair	12.5 (6.8 to 18.2)	<.001	−0.3 (−5.4 to 4.9)	.92
Poor	16.3 (6.9 to 25.6)	.001	−4.0 (−12.9 to 4.9)	.38
Employed				
Yes	1 [Reference]		1 [Reference]	
Retired	0.9 (−3.8 to 5.6)	.72	1.4 (−2.8 to 5.6)	.51
Unemployed	2.6 (−0.9 to 6.1)	.14	2.4 (−0.6 to 5.3)	.12
Family income, US $				
<30 000	1 [Reference]		1 [Reference]	
30 000-49 999	2.7 (−1.2 to 6.5)	.18	−1.3 (−4.7 to 2.0)	.44
50 000-79 999	3.1 (−0.8 to 7.1)	.12	−1.6 (−5.0 to 1.8)	.37
80 000-99 999	0.9 (−4.2 to 5.9)	.73	1.0 (−3.3 to 5.2)	.66
100 000-149 999	2.1 (−2.8 to 7.0)	.39	−2.6 (−7.0 to 1.7)	.24
150 000-199 999	2.6 (−4.5 to 9.8)	.48	0.9 (−5.3 to 7.2)	.77
≥200 000	3.9 (−4.3 to 12.1)	.35	4.9 (−2.0 to 11.7)	.16
Chronic disease, No.				
0	1 [Reference]		1 [Reference]	
1	0.9 (−2.5 to 4.3)	.62	1.4 (−1.6 to 4.3)	.36
2	4.0 (−0.4 to 8.5)	.08	1.0 (−2.9 to 5.0)	.61
>2	6.8 (1.5 to 12.1)	.01	1.2 (−3.5 to 6.0)	.62
Not sure	0.0 (−6.7 to 6.7)	.99	−3.4 (−9.5 to 2.7)	.28
Has primary care				
Yes	1 [Reference]		1 [Reference]	
No	−1.8 (−5.5 to 1.9)	.35	−2.6 (−5.9 to 0.8)	.13
Unsure	−4.9 (−12.2 to 2.4)	.19	2.8 (−3.0 to 8.7)	.34
Visits in past 6 mos				
Physician visit	0.1 (−0.5 to 0.7)	.82	0.1 (−0.4 to 0.7)	.60
ED visit	−0.9 (−2.8 to 1.0)	.34	−0.5 (−2.1 to 1.1)	.53
Hospital admissions in past 6 mos	−6.5 (−9.1 to −3.9)	<.001	−2.5 (−4.2 to −0.8)	.005
Global health care rating in past 6 mos, 0-10 points	0.5 (−0.2 to 1.2)	.19	−0.2 (−0.8 to 0.4)	.53

Abbreviations: ED, emergency department; GED, general educational development.

^a^ A marginal effect signifies that between 1 woman and 1 man who were otherwise similar in characteristics, for example, the man’s probability of a correct diagnosis would be 9.4 percentage points lower than the woman’s (the referent). For corresponding adjusted odds ratios, see eTable 5 in the Supplement.

^b^ Age cutoffs were chosen to protect the identity at the fringes of age and to distinguish between older and younger adults, as they may vary in sophistication in using the internet and will likely vary in frequency of contact with the health system.

Factors associated with correct triage were less consistent. Characteristics that were significantly associated with an increased rate of correct triage were age of 50-59 years (5.9 [95% CI, 1.8-10.0] percentage points more than those <30 years; *P* = .005), female sex (4.5 [95% CI, 2.3-6.8] percentage points more than for male sex; *P* < .001), and White race/ethnicity (9.7 [95% CI, 5.2-14.2] percentage points more than for Black race/ethnicity; *P* < .001).

## Discussion

In a given year in the US, almost two-thirds of adults use the internet to search for health information and roughly one-third of adults have used the internet specifically for self-diagnosis, trying to discover an underlying cause to a health problem that they or their family members may have.^17^ Among a nationally representative sample of survey participants asked to diagnose and select a triage for a clinical vignette, we observed that the use of the internet was associated with modest but significant improvements in diagnosis, but we observed no association with triage or anxiety. Although the perceived harm of an internet search for health information may be unfounded, the potential benefits are also currently minimal.

This work builds on others in the literature. Similar to the present study, research by Martin and colleagues^18^ demonstrated that performing an internet search among patients in an emergency department waiting room was not associated with increased anxiety. Wang and colleagues^19^ also noted that observational studies reported mixed results with respect to the association between an internet search and patient anxiety.

Results of this survey study challenge the common belief among clinicians and policy makers that using the internet to search for health information is harmful. We found that performing an internet search was associated with improved diagnosis. One potential reason for this disconnect is that over time, search engines have tried to direct people to higher-quality health information. For example, several search engines have their own built-in health information curated by major medical centers,^20^ and in this study, almost half of the respondents believed such information was the most helpful. In this study, only a small percentage of respondents used social media or forums, which may have a lower quality of information. We also found that performing an internet search was not associated with selection of a more aggressive triage option or with increased anxiety. That is, we found no evidence of the hypothetical scenario of patients believing they were having a heart attack and calling 911 in response.

These results could be framed quite differently. Although it was associated with no harm, any benefit of an internet search was small. A recent systematic review found an overall low level of quality of online health information, which might explain the modest association.^21^ Websites or applications specifically designed to help people diagnose and triage themselves may be more helpful. Previous evaluations of older tools (called symptom checkers) demonstrated that their performance was mixed, but newer tools use artificial intelligence and may be more beneficial.^13,14,22,23,24,25^

Another explanation for the small before-after changes that we observed is anchoring. Only a small fraction of respondents changed their diagnosis or triage decision after the internet search. Consistent with the theory of reinforcement seeking, internet searchers may simply look for information to justify their initial decision rather than being open to all recommendations.^26^

With or without an internet search, roughly three-quarters of participants were able to identify the severity of the situation and when to seek care. Participants were more accurate when a case was more severe: almost 9 in 10 emergent cases were triaged appropriately. These results were reassuring, although it is important to recognize that an incorrect triage occurred in 1 in 10 emergent cases. How this rate of triage inaccuracy compares with the triage inaccuracy rate for nurse triage lines and how it is associated with patient outcome remain unclear.

In contrast to triage, we found that participants were much worse at diagnosis, responding correctly only about half the time. Sociodemographic differences in diagnostic accuracy were observed, with adults older than 40 years, women, and those with more health care experience performing significantly better. Consistent with previous work demonstrating that baseline knowledge was associated with improved diagnostic accuracy,^11^ results of the present study implied that lived experience (lower perceived health status, more comorbidities, and older age) seemed to assist participants with triage and diagnosis. Lived experience may also explain better performance by women because they, in general, experience more health care and may make more decisions for their family to seek out care.^27^

### Limitations

This study has limitations. First, we tested responses using simulated cases. Survey participants may respond differently or perform an internet search differently if they themselves or their family members were actually experiencing the symptoms. However, the anxiety levels reported by respondents increased as case acuity increased, suggesting that they were internalizing the cases appropriately. Participants also appeared to take the task seriously, spending about 12 minutes per case, approximately 3 times longer than what other studies have reported for similar in-person tasks for personally experienced symptoms.^18^ Second, the study has generalizability concerns given that the sample was obtained through an online platform and was fully conducted online. However, the sample was representative of the national population in terms of sex, age, and census region. The online platform also ensured that participants used the internet and allowed us to leverage its built-in quality controls, which may not have been available with in-person testing.

Third, the study may have lacked power in some of the variables to detect statistically significant differences, although this does not change the overall findings. Fourth, the vignette validation method relied on a convenience sample of physicians, and the physicians were not entirely in agreement with our choices. However, we selected multiple experienced physicians, and the accuracy rates among the physicians were more than 90% for all of the vignettes.

## Conclusions

We found that, among survey participants, using the internet to search for health information was associated with small increases in diagnostic accuracy. However, we observed no association between an internet search for health information and triage accuracy.

## Notes

Supplement. **eTable 1.** Clinical Case Vignettes **eTable 2.** Exact Triage and First Correct Diagnosis Before and After Search **eTable 3.** Dichotomized Triage Accuracy, Stratified by Participant Characteristics **eTable 4.** Any Correct Diagnosis Accuracy, Stratified by Participant Characteristics **eTable 5.** Predictors of Any Correct Diagnosis and Dichotomized Triage Before and After Search **eTable 6.** Dichotomized Triage, Any Correct Diagnosis, Anxiety, and Confidence Before and After Search **eTable 7.** Search Content Characteristics **eFigure.** Anchoring and Flipping
