# Supplementary material for: Assessment of Diagnosis and Triage in Validated Case Vignettes Among Nonphysicians Before and After Internet Search
Source: JAMA Netw Open. 2021 Mar 29;4(3):e213287. doi: 10.1001/jamanetworkopen.2021.3287 (PMC8008286; doi:10.1001/jamanetworkopen.2021.3287)
Supplement: Supplement. — eTable 1. Clinical Case Vignettes eTable 2. Exact Triage and First Correct Diagnosis Before and After Search eTable 3. Dichotomized Triage Accuracy, Stratified by Participant Characteristics eTable 4. Any Correct Diagnosis Accuracy, Stratified by Participant Characteristics eTable 5. Predictors of Any Correct Diagnosis and Dichotomized Triage Before and After Search eTable 6. Dichotomized Triage, Any Correct Diagnosis, Anxiety, and Confidence Before and After Search eTable 7. Search Content Characteristics eFigure. Anchoring and Flipping [file jamanetwopen-e213287-s001.pdf]

Supplementary Online Content

Levine DM, Mehrotra A. Assessment of diagnosis and triage in validated case vignettes among nonphysicians before and after internet search. *JAMA Netw Open*. 2021;4(3):e213287. doi:10.1001/jamanetworkopen.2021.3287

eTable 1. Clinical Case Vignettes

eTable 2. Exact Triage and First Correct Diagnosis Before and After Search

eTable 3. Dichotomized Triage Accuracy, Stratified by Participant Characteristics

eTable 4. Any Correct Diagnosis Accuracy, Stratified by Participant Characteristics

eTable 5. Predictors of Any Correct Diagnosis and Dichotomized Triage Before and After Search

eTable 6. Dichotomized Triage, Any Correct Diagnosis, Anxiety, and Confidence Before and After Search

eTable 7. Search Content Characteristics

eFigure. Anchoring and Flipping

This supplementary material has been provided by the authors to give readers additional information about their work.

eTable 1. Clinical Case Vignettes

Case #	Correct Diagnosis	Correct Triage	Current Problem	Additional Details
1	Liver failure	Emergent	Confused and sleepy for several hours	48-year-old female<br>Can't answer where she is or what her name is (but she normally can)<br>Takes Tylenol all the time for migraine, but recently took more
2	Appendicitis	Emergent	Sudden severe abdominal pain	12-year-old female<br>Temperature 104 F (40 C)<br>Has nausea, vomiting, and diarrhea
3	Heart attack	Emergent	Chest pain for 1 day	64-year-old male<br>Pain is in middle of chest under breast bone<br>Sweating and having trouble breathing
4	Kidney stones	Emergent	Left-sided pain between armpit and hip for 1 hour	45-year-old male<br>Severe pain<br>Pain starts on left side and travels to groin<br>Has nausea and vomiting<br>Squirming in pain
5	Meningitis	Emergent	Headache for 3 days	18-year-old male<br>Has fever 102<br>Neck stiff<br>Light bothers him
6	Pulmonary embolism	Emergent	Trouble breathing for 30 minutes	65-year-old male<br>Has chest pain that worsens when he takes a breath<br>Had surgery last week<br>Has swelling and pain in his left calf
7	Rocky Mountain Spotted Fever	Emergent	Fever and rash for 2 days	8-year-old male<br>Rash is worse on the ankles and wrists<br>Has joint pain and headache<br>Was camping recently
8	Stroke	Emergent	Right-sided weakness and difficulty speaking for 10 minutes	70-year-old male<br>Can't use his right arm<br>Has high blood pressure and abnormal heart rhythm<br>Has nausea and vomiting
9	Tetanus	Emergent	Painful muscle spasms for 1 day	65-year-old male<br>Having a hard time opening his mouth

©2021 Levine DM et al. *JAMA Network Open.*

				Cut himself while gardening<br>He is restless and irritable
10	COPD exacerbation	Emergent	Trouble breathing for 3 days	67-year-old female<br>Has lung disease and smoked cigarettes<br>Increased cough with green phlegm<br>Can't speak in complete sentences
11	Asthma exacerbation	Emergent	Trouble breathing for 3 days	27-year-old female<br>Recent cold<br>Wheezing and coughing when sitting<br>Trouble breathing when walking around house<br>Has asthma<br>Inhalers don't help her anymore
12	Heart failure exacerbation	Emergent	Trouble breathing for 3 days	78-year-old female<br>Ate very salty foods recently<br>Gaining weight and legs are swollen<br>Had a heart attack several years ago<br>Trouble breathing when sitting on the couch
13	Cellulitis	1 day	Front of left leg is red and hurts for one day	45-year-old man<br>Redness started quickly over a day<br>He thinks he has a high temperature, but he didn't check<br>Leg is swollen<br>Leg hurts to touch it, but he didn't cut it or bump it that he remembers
14	Strep throat	1 day	Fever and sore throat for 2 hours	7-year-old female<br>White stuff in back of throat<br>Painful front of the neck<br>No cough, no congestion<br>Temperature 102
15	Asthma exacerbation	1 day	Trouble breathing for 3 days	27-year-old female<br>Recent cold<br>Wheezing and coughing, especially at night<br>Has asthma<br>Inhalers only help for a couple of hours
16	COPD exacerbation	1 day	Trouble breathing for 3 days	67-year-old female<br>Has lung disease and smoked cigarettes<br>Increased cough with green phlegm<br>Using inhaler every 6 hours

©2021 Levine DM et al. *JAMA Network Open.*

17	Deep vein thrombosis	1 day	Right leg pain for 2 days	65-year-old female<br>Right leg is swollen, red, and painful<br>Was in hospital last week for pneumonia<br>Has mild heart disease
18	Hemolytic uremic syndrome	1 day	Belly pain and diarrhea for 7 days	4-year-old male<br>Nothing unusual in diet though did have a hamburger at a cookout 3 days before pain started<br>Has fever<br>Diarrhea may have blood in it
19	Malaria	1 day	Fever and chills for 5 days	18-year-old male<br>Has fever of 103.1F<br>Recently returned from Africa
20	Pneumonia	1 day	Cough and fever for 3 days	65-year-old male<br>Cough brings up green phlegm<br>Breathing fast and shallow
21	Mononucleosis	1 day	Fever, sore throat, enlarged lymph nodes for one week	16-year-old female<br>Trouble swallowing<br>Extremely tired
22	Salmonella	1 day	Vomiting, diarrhea, and belly pain for 18 hours	14-year-old male<br>Had diarrhea 10 times in 18 hours<br>No blood in diarrhea<br>Has temperature of 103.1F<br>Attended a picnic recently and ate undercooked chicken
23	Shingles	1 day	Pain on right side of chest for 5 days	77-year-old male<br>Pain was first and was then followed by rash in same area as pain<br>Feels unwell in general<br>Rash becomes clear pockets of fluid on top of skin after 3 days
24	Urinary tract infection	1 day	Painful urination for 2 days	26-year-old female<br>Has urgent need to pee<br>Recent sexual activity<br>No fever, but some back pain
25	Acne	1 week	Red bumps on forehead for 4 weeks	17-year-old male<br>Bumps come and go and then come in new places on face
26	Acute sinusitis	1 week	Feels congested and face pain for 15 days	35-year-old female<br>Has green nose discharge<br>Has a fever again after thinking she was getting better

©2021 Levine DM et al. *JAMA Network Open.*

27	Peptic ulcer disease	1 week	Upper abdominal pain for 2 months	40-year-old male<br>Feels like a dull, gnawing ache<br>Wakes him up at night<br>Better when drinking milk and eating
28	Vertigo	1 week	Sudden episodes of dizziness for 1 month	65-year-old female<br>Triggered by tilting her head back to look up<br>Each attack lasts about 30 seconds<br>No hearing problems or weakness
29	Diabetes	1 week	Thirsty and peeing a lot for 4 weeks	52-year-old male<br>Feels tired all the time<br>Has blurry vision on and off
30	Hemorrhoids	1 week	Blood on toilet paper for 1 week	68-year-old female<br>Stool is hard and must strain when using the bathroom<br>No weight loss, belly pain, or fever<br>Stool is not red or black
31	Osteoarthritis	1 week	Left knee pain for 4 months	62-year-old male<br>Worse after walking, better when resting<br>Sometimes swells up<br>Never hit knee against anything or fell
32	Depression	1 week	Feeling down for weeks	32-year-old male<br>Wakes up early and cannot go back to sleep<br>Having difficulty concentrating at work<br>Cannot seem to stop worrying about his family
33	Obstructive sleep apnea	1 week	Tired for 2 months	42-year-old female<br>Overweight<br>Falls asleep during the day<br>Partner complains about snoring at night
34	Heart failure exacerbation	1 week	Trouble breathing for 7 days	78-year-old female<br>Slowly gaining weight<br>Had a heart attack several years ago<br>Comfortable when sitting or walking slowly, but has trouble when walking up stairs
35	Hypothyroidism	1 week	Tired for 2 months	39-year-old female<br>Has dry skin, weight gain, and constipation<br>Feels very cold when others are hot

©2021 Levine DM et al. *JAMA Network Open.*

36	Gastroesophageal reflux disease	1 week	Upper abdominal pain for 2 months	40-year-old male<br>Worse with spicy and fried foods<br>Worse if he eats late at night before sleeping<br>Voice is becoming hoarse
37	Common cold	Self-care	Runny nose for 12 days	34-year-old female<br>No fever<br>Had also had a sore throat and cough<br>No other medical problems
38	Viral conjunctivitis	Self-care	Red, irritated eye for 3 days	14-year-old male<br>Spread from right to left<br>Has watery eye discharge, but no pain<br>Has a stuffy nose
39	Viral pharyngitis	Self-care	Sore throat for 2 days	26-year-old male<br>Has a headache<br>Has a cough<br>No fever
40	Allergic rhinitis	Self-care	Congestion for many years	22-year-old male<br>Worse during spring season<br>Has sneezing and nose itching<br>Has eye itching and tearing
41	Musculoskeletal low back pain	Self-care	Back pain for 3 weeks	35-year-old male<br>Pain started after shoveling snow<br>No leg pain or weakness<br>No fever or weight loss
42	Bee sting without anaphylaxis	Self-care	Swollen and tender forehead at site of bee sting for 1 hour	9-year-old male<br>Stopped crying after 15 minutes<br>Tongue looks normal<br>No wheezing or breathing problems
43	Canker sore	Self-care	Mouth sores that come back over several years	17-year-old male<br>Has 5 of them in his mouth<br>No other sores anywhere else<br>Takes no drugs or medicines
44	Candidal yeast infection	Self-care	White stuff coming out of vagina for 2 days	40-year-old female<br>Vagina also itchy<br>Doesn't hurt to pee<br>No abdominal pain<br>No fever
45	Eczema	Self-care	Dry itchy skin in front of elbows and behind knees for years	12-year-old female<br>Brother has asthma<br>Recently told she has egg and milk allergies

©2021 Levine DM et al. *JAMA Network Open.*

46	Stye	Self-care	Painful swollen right eyelid for 1 day	30-year-old male<br>Pain is at edge of eyelid<br>Hurts to touch it<br>No change in vision
47	Influenza	Self-care	Fever, headache, and cough for 4 days	30-year-old female<br>Came on very suddenly<br>Feels weak all over<br>Temperature 102.5 at first, but not now
48	Poison Ivy	Self-care	Itchy rash on left hand for 1 day	12-year-old male<br>Came on hours after playing in woods<br>Rash red and warm<br>No fever

©2021 Levine DM et al. *JAMA Network Open.*

eTable 2. Exact Triage and First Correct Diagnosis Before and After Search

	Before search (n=5000)	After search (n=5000)	Difference[a]
	Mean % correct (95% confidence interval)		
**Triage, exact**			
All cases	47.3 (45.9,48.7)	46.1 (44.7,47.5)	-1.2 (-2.5,0.1)
Emergent cases	57.5 (54.8,60.2)	55.4 (52.7,58.2)	-2.0 (-4.5,0.4)
1-day cases	50.0 (47.2,52.9)	48.7 (45.9,51.5)	-1.3 (-4.2,1.5)
1-week cases	57.3 (54.4,60.2)	56.1 (53.2,59.0)	-1.2 (-4.0,1.6)
Self-care cases	25.4 (23.0,27.8)	25.1 (22.7,27.5)	-0.3 (-2.4,1.8)
**Diagnosis, 1st correct**			
All cases	36.8 (35.5,38.1)	39.5 (38.1,40.8)	2.7 (1.5,3.9)
Emergent cases	27.6 (25.1,30.0)	30 (27.5,32.5)	2.4 (0.3,4.6)
1-day cases	25.3 (22.9,27.8)	27.3 (24.8,29.8)	2.0 (-0.2,4.2)
1-week cases	41.1 (38.4,43.8)	45.8 (43,48.6)	4.7 (2.1,7.3)
Self-care cases	52.9 (50.2,55.7)	54.5 (51.8,57.3)	1.6 (-1.1,4.2)

[a] After search minus before search: positive values indicate searcher more effective after searching than before searching

©2021 Levine DM et al. *JAMA Network Open.*

eTable 3. Dichotomized Triage Accuracy, Stratified by Participant Characteristics

	Before search (n=5000)	After search (n=5000)	Difference[a]
	Mean % correct (95% confidence interval)		
All cases	74.5 (73.3,75.7)	74.1 (72.9,75.3)	-0.4 (-1.4,0.6)
**GENDER**			
Female	76.9 (75.3,78.5)	77.3 (75.7,79.0)	0.4 (-0.9,1.8)
Male	72.0 (70.2,73.7)	70.9 (69.0,72.7)	-1.1 (-2.5,0.3)
Other	Suppress	Suppress	Suppress
**RACE/ETHNICITY**			
Non-Hispanic White	76.1 (74.7,77.4)	75.7 (74.4,77.1)	-0.3 (-1.4,0.7)
Hispanic	68.9 (63.8,74.0)	70.1 (65.1,75.2)	1.3 (-3.5,6.1)
Non-Hispanic Black	63.4 (58.6,68.1)	66.1 (61.5,70.7)	2.7 (-1.2,6.6)
Non-Hispanic Asian	74.8 (69.9,79.6)	70.9 (65.8,76.0)	-3.9 (-8.2,0.4)
Non-Hispanic Other or Multiple	75.3 (68.4,82.3)	70.0 (62.6,77.4)	-5.3 (-11.5,0.8)
**CENSUS REGION**			
Northeast	74.4 (71.6,77.2)	73.8 (71.0,76.6)	-0.6 (-2.8,1.5)
Midwest	74.3 (71.8,76.9)	75.1 (72.6,77.6)	0.8 (-1.3,2.9)
South	76.2 (74.2,78.2)	75.0 (72.9,77.0)	-1.3 (-2.9,0.4)
West	72.0 (69.4,74.6)	72.1 (69.5,74.7)	0.1 (-2.1,2.3)
**PARTNER STATUS**			
Married/partnered	75.2 (73.6,76.8)	75.2 (73.6,76.8)	0.0 (-1.3,1.2)
Not married/partnered	73.5 (71.6,75.4)	72.7 (70.8,74.6)	-0.8 (-2.4,0.8)
**EDUCATION**			
<High school	66.0 (56.7,75.3)	67.0 (57.8,76.2)	1.0 (-7.5,9.4)
High school/GED	71.8 (69.1,74.6)	71.5 (68.8,74.3)	-0.3 (-2.5,1.9)
Some college	75.4 (73.4,77.5)	74.9 (72.8,77.0)	-0.5 (-2.2,1.2)
Bachelor's degree	75.1 (72.8,77.3)	74.6 (72.3,76.8)	-0.5 (-2.4,1.4)
>Bachelor's	76.2 (73.1,79.4)	76.2 (73.1,79.4)	0.0 (-2.4,2.4)
**HEALTH INSURANCE COVERAGE**			
Uninsured	74.5 (70.4,78.6)	71.3 (67.1,75.6)	-3.2 (-6.8,0.4)
Medicare	73.6 (71.0,76.1)	73.4 (70.8,76.0)	-0.2 (-2.1,1.7)
Medicaid	69.3 (65.4,73.3)	71.8 (67.9,75.6)	2.4 (-1.0,5.9)
Both Medicare and Medicaid	66.3 (59.5,73.1)	65.8 (58.9,72.6)	-0.5 (-6.2,5.2)
Private/employer-based	77.1 (75.4,78.7)	76.9 (75.2,78.6)	-0.2 (-1.6,1.2)
I'm not sure.	68.6 (62.2,75.0)	64.2 (57.6,70.8)	-4.4 (-9.9,1.1)
**PERCEIVED HEALTH STATUS**			
Excellent	71.4 (68.1,74.8)	69.7 (66.3,73.1)	-1.7 (-4.5,1.0)
Very good	73.7 (71.7,75.8)	73.4 (71.3,75.4)	-0.3 (-2.0,1.4)
Good	77.4 (75.5,79.4)	77.6 (75.7,79.6)	0.2 (-1.3,1.8)
Fair	72.7 (69.2,76.3)	72.1 (68.5,75.7)	-0.7 (-3.6,2.3)

©2021 Levine DM et al. *JAMA Network Open.*

Poor	68.7 (60.7,76.7)	68.7 (60.7,76.7)	0.0 (-6.8,6.8)
**EMPLOYED**			
Yes	74.0 (72.4,75.6)	73.9 (72.3,75.5)	-0.1 (-1.4,1.2)
Retired	76.3 (73.7,79.0)	75.3 (72.6,78.0)	-1.0 (-3.0,1.0)
Unemployed	74.1 (71.5,76.8)	73.7 (71.0,76.3)	-0.5 (-2.8,1.9)
**FAMILY INCOME**			
<$30,000	73.0 (70.6,75.5)	73.7 (71.2,76.1)	0.6 (-1.5,2.8)
$30,000-$49,999	73.8 (71.0,76.5)	72.9 (70.1,75.6)	-0.9 (-3.1,1.3)
$50,000-$79,999	74.5 (72.0,77.0)	73.9 (71.3,76.4)	-0.7 (-2.7,1.4)
$80,000-$99,999	77.9 (74.4,81.4)	76.2 (72.6,79.8)	-1.7 (-4.8,1.4)
$100,000-$149,999	73.6 (70.3,77.0)	73.3 (70.0,76.7)	-0.3 (-2.9,2.3)
$150,000-$199,999	76.4 (70.9,82.0)	77.3 (71.8,82.8)	0.9 (-3.2,5.0)
$200,000 or more	79.7 (73.2,86.3)	79.7 (73.2,86.3)	0.0 (-4.6,4.6)
**CHRONIC DISEASES**			
0	73.6 (71.9,75.3)	73.3 (71.6,75.0)	-0.3 (-1.7,1.1)
1	75.5 (72.9,78.1)	75.4 (72.8,78.0)	-0.1 (-2.2,2.0)
2	76.5 (73.1,79.9)	76.0 (72.6,79.4)	-0.5 (-3.0,2.0)
>2	76.0 (72.3,79.6)	75.4 (71.7,79.1)	-0.6 (-3.5,2.3)
Not sure	70.3 (63.6,76.9)	68.6 (61.9,75.4)	-1.6 (-7.6,4.3)
**HAS PRIMARY CARE**			
Yes	74.9 (73.6,76.3)	75.2 (73.9,76.6)	0.3 (-0.8,1.4)
No	72.5 (69.5,75.4)	69.8 (66.7,72.9)	-2.7 (-5.2,-0.1)
Unsure	74.0 (67.4,80.6)	69.9 (63.0,76.8)	-4.0 (-10.2,2.1)

©2021 Levine DM et al. *JAMA Network Open.*

eTable 4. Any Correct Diagnosis Accuracy, Stratified by Participant Characteristics

	Before search (n=5000)	After search (n=5000)	Difference[a]
	Mean % correct (95% confidence interval)		
**All cases**	49.8 (48.4,51.2)	54.0 (52.6,55.4)	4.2 (3.1,5.3)
**GENDER**			
Female	55.6 (53.7,57.5)	59.2 (57.3,61.1)	3.6 (2.0,5.1)
Male	43.7 (41.7,45.7)	48.6 (46.6,50.6)	4.9 (3.4,6.3)
Other	Suppress	Suppress	Suppress
**RACE/ETHNICITY**			
Non-Hispanic White	52.9 (51.3,54.5)	56.8 (55.2,58.4)	3.9 (2.7,5.2)
Hispanic	42.1 (36.7,47.6)	43.4 (37.9,48.9)	1.3 (-2.5,5.0)
Non-Hispanic Black	39.1 (34.3,43.9)	44.1 (39.2,48.9)	5.0 (1.5,8.4)
Non-Hispanic Asian	33.3 (28.0,38.6)	43.0 (37.5,48.6)	9.7 (5.6,13.8)
Non-Hispanic Other or Multiple	50.0 (41.9,58.1)	54.0 (45.9,62.1)	4.0 (-3.4,11.4)
**CENSUS REGION**			
Northeast	47.3 (44.1,50.4)	53.2 (50.0,56.3)	5.9 (3.5,8.3)
Midwest	53.3 (50.4,56.2)	55.3 (52.4,58.2)	2.0 (-0.2,4.2)
South	50.7 (48.4,53.1)	54.7 (52.4,57.0)	3.9 (2.1,5.8)
West	47.0 (44.1,49.8)	52.3 (49.5,55.2)	5.4 (3.2,7.6)
**PARTNER STATUS**			
Married/partnered	51.2 (49.4,53.1)	55.2 (53.4,57.1)	4.0 (2.6,5.4)
Not married/partnered	47.9 (45.8,50.0)	52.4 (50.3,54.5)	4.5 (2.8,6.1)
**EDUCATION**			
<High school	42.7 (33.0,52.4)	50.5 (40.7,60.3)	7.8 (0.7,14.8)
High school/GED	46.8 (43.8,49.8)	49.7 (46.6,52.7)	2.9 (0.5,5.3)
Some college	51.4 (49.1,53.8)	55.5 (53.2,57.9)	4.1 (2.3,5.9)
Bachelor's degree	49.6 (47.0,52.2)	54.8 (52.2,57.4)	5.2 (3.2,7.1)
>Bachelor's	51.7 (48.0,55.4)	55.7 (52.0,59.3)	4.0 (1.1,6.8)
**HEALTH INSURANCE COVERAGE**			
Uninsured	47.2 (42.5,51.8)	54.0 (49.3,58.6)	6.8 (2.8,10.7)
Medicare	48.0 (45.1,50.9)	49.9 (47.0,52.8)	1.8 (-0.1,3.8)
Medicaid	48.8 (44.5,53.0)	53.8 (49.6,58.1)	5.0 (1.8,8.3)
Both Medicare and Medicaid	44.4 (37.2,51.6)	42.2 (35.1,49.4)	-2.1 (-6.9,2.6)
Private/employer-based	52.5 (50.6,54.5)	57.4 (55.4,59.3)	4.8 (3.3,6.4)
I’m not sure.	39.7 (32.9,46.5)	47.5 (40.6,54.5)	7.8 (3.0,12.7)
**PERCEIVED HEALTH STATUS**			
Excellent	39.9 (36.2,43.5)	43.3 (39.6,47.0)	3.5 (1.0,6.0)
Very good	49.0 (46.7,51.4)	54.2 (51.9,56.5)	5.2 (3.4,6.9)
Good	51.7 (49.4,54.0)	55.7 (53.4,58.0)	4.0 (2.2,5.9)
Fair	55.9 (51.9,59.8)	58.0 (54.1,62.0)	2.1 (-1.1,5.4)
Poor	58.0 (49.5,66.6)	64.9 (56.6,73.2)	6.9 (0.7,13)

©2021 Levine DM et al. *JAMA Network Open.*

**EMPLOYED**			
Yes	48.6 (46.8,50.4)	53.1 (51.3,54.9)	4.5 (3.1,5.8)
Retired	54.0 (51.0,57.1)	55.2 (52.2,58.3)	1.2 (-1.1,3.5)
Unemployed	49.0 (46.0,52.0)	55.3 (52.3,58.3)	6.3 (3.8,8.8)
**FAMILY INCOME**			
<$30,000	47.9 (45.2,50.7)	51.4 (48.7,54.2)	3.5 (1.4,5.6)
$30,000-$49,999	51.1 (47.9,54.2)	54.2 (51.1,57.3)	3.1 (0.8,5.5)
$50,000-$79,999	51.5 (48.7,54.4)	55.7 (52.8,58.5)	4.1 (2.0,6.3)
$80,000-$99,999	49.8 (45.6,54.1)	53.2 (48.9,57.4)	3.4 (-0.1,6.8)
$100,000-$149,999	49.5 (45.7,53.4)	55.2 (51.3,59.0)	5.6 (2.5,8.7)
$150,000-$199,999	47.6 (41.0,54.1)	57.3 (50.8,63.8)	9.8 (5.3,14.2)
$200,000 or more	48.0 (39.8,56.1)	54.1 (45.9,62.2)	6.1 (0.0,12.1)
**CHRONIC DISEASES**			
0	47.0 (45.1,48.9)	51.4 (49.5,53.3)	4.4 (2.9,5.9)
1	49.6 (46.6,52.6)	53.4 (50.4,56.4)	3.8 (1.5,6.0)
2	54.3 (50.4,58.2)	59.6 (55.8,63.5)	5.3 (2.3,8.4)
>2	59.8 (55.6,64.0)	62.5 (58.3,66.7)	2.7 (-0.3,5.7)
Not sure	47.6 (40.3,54.8)	51.9 (44.6,59.2)	4.3 (-2.1,10.7)
**HAS PRIMARY CARE**			
Yes	50.9 (49.4,52.5)	55.2 (53.7,56.8)	4.3 (3.1,5.4)
No	46.5 (43.2,49.9)	50.8 (47.5,54.2)	4.3 (1.5,7.0)
Unsure	39.9 (32.5,47.3)	42.2 (34.8,49.6)	2.3 (-3.5,8.1)

©2021 Levine DM et al. *JAMA Network Open.*

eTable 5. Predictors of Any Correct Diagnosis and Dichotomized Triage Before and After Search

	Correct Diagnosis		Correct Triage	
	aOR	p-value	aOR	p-value
**AGE**				
<30	Referent		Referent	
30-39	1.04 (0.88,1.24)	0.626	1.02 (0.85,1.23)	0.799
40-49	1.24 (1.03,1.48)	0.020	1.17 (0.96,1.43)	0.126
50-59	1.32 (1.08,1.61)	0.006	1.38 (1.10,1.74)	0.005
60-69	1.19 (0.94,1.50)	0.152	1.13 (0.87,1.47)	0.363
>=70	1.25 (0.93,1.68)	0.137	1.21 (0.87,1.67)	0.254
**SEX**				
Female	Referent		Referent	
Male	0.67 (0.61,0.75)	<0.001	0.79 (0.70,0.89)	<0.001
**RACE/ETHNICITY**				
Non-Hispanic White	Referent		Referent	
Non-Hispanic Black	0.67 (0.55,0.82)	<0.001	0.62 (0.50,0.76)	<0.001
Hispanic	0.77 (0.61,0.96)	0.022	0.86 (0.68,1.08)	0.196
Non-Hispanic Asian	0.54 (0.43,0.68)	<0.001	0.90 (0.70,1.15)	0.398
Non-Hispanic Other or Multiple	1.02 (0.74,140)	0.927	0.99 (0.70,1.40)	0.943
**CENSUS REGION**				
Northeast	Referent		Referent	
Midwest	1.02 (0.87,1.20)	0.816	0.97 (0.81,1.17)	0.779
South	1.03 (0.88,1.20)	0.719	1.07 (0.90,1.27)	0.468
West	0.95 (0.81,1.13)	0.577	0.85 (0.71,1.03)	0.090
**PARTNER STATUS**				
Married/partnered	Referent		Referent	
Not married/partnered	1.02 (0.90,1.15)	0.784	1.03 (0.90,1.17)	0.716
**EDUCATION**				
<High school	Referent		Referent	
High school/GED	1.04 (0.71,1.53)	0.833	1.17 (0.79,1.74)	0.436
Some college	1.23 (0.84,1.80)	0.285	1.37 (0.93,2.03)	0.115
Bachelor's degree	1.26 (0.85,1.86)	0.246	1.37 (0.92,2.05)	0.123
>Bachelor's	1.36 (0.91,2.05)	0.139	1.43 (0.93,2.19)	0.102
**HEALTH INSURANCE COVERAGE**				
Uninsured	Referent		Referent	
Medicare	0.68 (0.53,0.88)	0.003	0.83 (0.63,1.09)	0.171
Medicaid	0.88 (0.68,1.13)	0.323	0.79 (0.60,1.04)	0.094
Both Medicare and Medicaid	0.66 (0.46,0.94)	0.021	0.68 (0.47,0.99)	0.043
Private/employer-based	0.99 (0.80,1.24)	0.960	1.06 (0.83,1.35)	0.655
I'm not sure.	0.77 (0.55,1.07)	0.121	0.69 (0.49,0.97)	0.034
**PERCEIVED HEALTH STATUS**				
Excellent	Referent		Referent	
Very good	1.35 (1.14,1.60)	0.001	1.05 (0.88,1.27)	0.578
Good	1.45 (1.21,1.74)	<0.001	1.31 (1.07,1.60)	0.008
Fair	1.69 (1.33,2.16)	<0.001	0.99 (0.76,1.28)	0.922
Poor	2.00 (1.33,3.00)	0.001	0.82 (0.54,1.26)	0.367
**EMPLOYED**				
Yes	Referent		Referent	
Retired	1.04 (0.85,1.27)	0.715	1.08 (0.86,1.36)	0.510
Unemployed	1.12 (0.96,1.30)	0.141	1.14 (0.97,1.34)	0.121

©2021 Levine DM et al. *JAMA Network Open.*

**FAMILY INCOME**				
<$30,000	Referent		Referent	
$30,000-$49,999	1.12 (0.95,1.32)	0.180	0.93 (0.78,1.12)	0.436
$50,000-$79,999	1.14 (0.97,1.35)	0.122	0.92 (0.76,1.10)	0.365
$80,000-$99,999	1.04 (0.84,1.29)	0.730	1.06 (0.83,1.34)	0.660
$100,000-$149,999	1.09 (0.89,1.35)	0.394	0.87 (0.69,1.09)	0.235
$150,000-$199,999	1.12 (0.83,1.51)	0.476	1.05 (0.74,1.49)	0.770
$200,000 or more	1.18 (0.83,1.67)	0.350	1.33 (0.87,2.04)	0.186
**CHRONIC DISEASES**				
0	Referent		Referent	
1	1.04 (0.90,1.20)	0.622	1.08 (0.92,1.27)	0.365
2	1.19 (0.98,1.43)	0.077	1.06 (0.85,1.31)	0.612
>2	1.33 (1.06,1.67)	0.013	1.07 (0.82,1.39)	0.619
Not sure	1.00 (0.76,1.33)	0.994	0.84 (0.62,1.14)	0.261
**HAS PRIMARY CARE**				
Yes	Referent		Referent	
No	0.93 (0.79,1.09)	0.346	0.87 (0.73,1.04)	0.122
Unsure	0.81 (0.60,1.11)	0.190	1.18 (0.84,1.65)	0.355
**PHYSICIAN VISITS** in last 6 months	1.00 (0.98,1.03)	0.824	1.01 (0.98,1.04)	0.596
**EMERGENCY ROOM VISITS** in last 6 months	0.96 (0.89,1.04)	0.343	0.97 (0.89,1.06)	0.529
**HOSPITAL ADMISSIONS** in last 6 months	0.76 (0.68,0.85)	<0.001	0.88 (0.80,0.96)	0.005
**GLOBAL HEALTH CARE RATING** in last 6 months (0-10)	1.02 (0.99,1.05)	0.189	0.99 (0.96,1.02)	0.529

Abbreviations: aOR, adjusted odds ratio; GED, general education development

©2021 Levine DM et al. *JAMA Network Open.*

eTable 6. Dichotomized Triage, Any Correct Diagnosis, Anxiety, and Confidence Before and After Search

	Before search (n=5000)	After search (n=5000)	Difference[a]
	Mean % correct (95% confidence interval)		
**Triage**			
All cases	74.5 (73.3,75.7)	74.1 (72.9,75.3)	-0.4 (-1.4,0.6)
Emergent cases	87.0 (85.2,88.9)	87.2 (85.4,89.1)	0.2 (-1.5,2.0)
1-day cases	81.3 (79.1,83.5)	79.5 (77.3,81.8)	-1.7 (-3.7,0.2)
1-week cases	60.4 (57.7,63.1)	60.4 (57.6,63.1)	-0.1 (-2.3,2.2)
Self-care cases	69.3 (66.7,71.8)	69.3 (66.8,71.9)	0.1 (-1.9,2.1)
**Diagnosis**			
All cases	49.8 (48.4,51.2)	54.0 (52.6,55.4)	4.2 (3.1,5.3)
Emergent cases	40.5 (37.8,43.2)	43.6 (40.9,46.4)	3.1 (1.0,5.3)
1-day cases	38.1 (35.4,40.9)	41.7 (38.9,44.4)	3.5 (1.5,5.6)
1-week cases	52.8 (50.1,55.6)	59.2 (56.5,62.0)	6.4 (4.1,8.7)
Self-care cases	67.4 (64.8,70.0)	71.2 (68.7,73.7)	3.7 (1.7,5.8)
**Anxiety**[b]			
All cases	3.3 (3.2,3.3)	3.3 (3.3,3.3)	0.05 (0.03,0.07)
Emergent cases	3.9 (3.8,3.9)	3.9 (3.9,4.0)	0.06 (0.03,0.10)
1-day cases	3.6 (3.5,3.6)	3.6 (3.5,3.6)	0.02 (-0.02,0.06)
1-week cases	3.1 (3.0,3.1)	3.1 (3.1,3.2)	0.07 (0.03,0.11)
Self-care cases	2.6 (2.5,2.6)	2.6 (2.6,2.7)	0.05 (0.01,0.09)
**Confidence in own rating**[b]			
All cases	3.8 (3.8,3.8)	3.8 (3.8,3.8)	-0.02 (-0.04,0.00)
Emergent cases	4.0 (4.0,4.1)	4.0 (3.9,4.0)	-0.02 (-0.07,0.02)
1-day cases	3.8 (3.8,3.9)	3.9 (3.8,3.9)	0.04 (-0.01,0.08)
1-week cases	3.7 (3.6,3.7)	3.7 (3.6,3.7)	-0.02 (-0.06,0.02)
Self-care cases	3.7 (3.7,3.8)	3.7 (3.6,3.7)	-0.08 (-0.12,-0.03)

[a] After search minus before search: positive values indicate searcher more effective after searching than before searching

[b] Likert scale, 1-5: not at all / slightly / moderately / highly / extremely

©2021 Levine DM et al. *JAMA Network Open.*

eTable 7. Search Content Characteristics

	Internet Searchers (n = 5000)
**Difficult to find useful information**, (95% CI)[a]	2.3 (2.3,2.3)
**Trust information found**, (95% CI)[a]	3.2 (3.2,3.3)
**Most helpful source**, n (%)	
Direct from search engine	2411 (48.2)
Health specialty site	2145 (42.9)
General information site	204 (4.1)
Social network site	73 (1.5)
News site	25 (0.5)
Forum	12 (0.2)
Other	130 (2.6)

[a] Likert scale, 1-5: not at all / slightly / moderately / highly / extremely

©2021 Levine DM et al. *JAMA Network Open.*

eFigure. Anchoring and Flipping

a) Diagnosis (1st correct). b) Triage (exact correct)

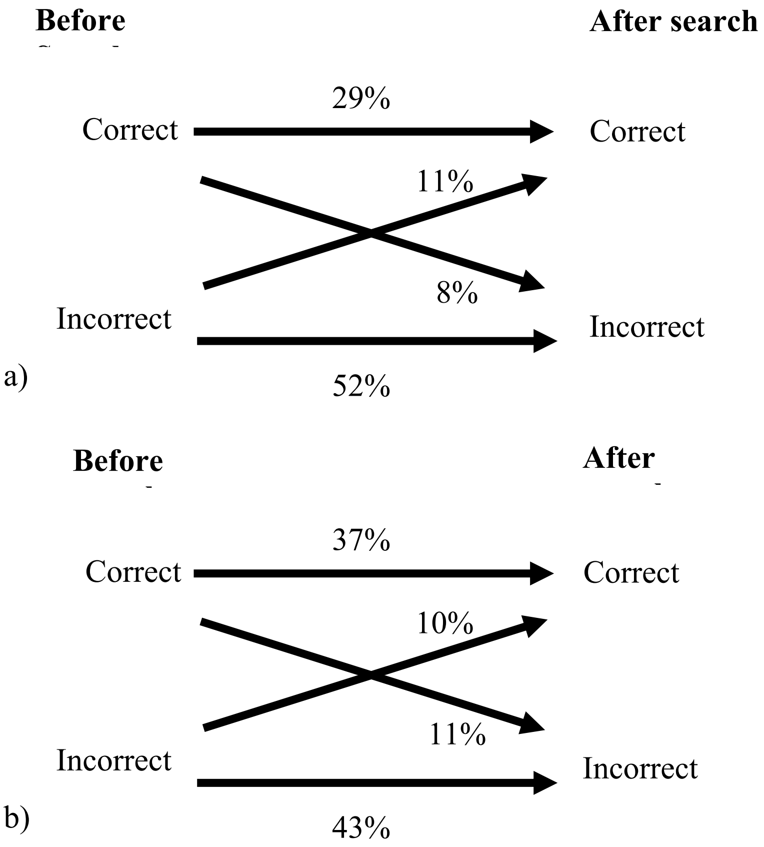

©2021 Levine DM et al. *JAMA Network Open.*
